# Sleep Symptoms and Polysomnographic Patterns of Obstructive Sleep Apnea in Obese Children

**Published:** 2016

**Authors:** Azita TAVASOLI, Shabnam JALILOLGHADR, Shiva LOTFI

**Affiliations:** 1 Pediatric Neurology Department, Ali-Asghar Children’s Hospital, Iran University of Medical Sciences, Tehran, Iran; 2 Pediatrics and Sleep Disorder Department, Qods Hospital, Qazvin University of Medical Sciences, Qazvin, Iran; 3 General Physicians, Qazvin University of Medical Sciences, Qazvin, Iran

**Keywords:** BEARS questionnaire, Polysomnography, Obstructive apnea, Obesity, Sleep apnea- hypopnea syndrome

## Abstract

**Objective:**

This study was conducted to investigate the sleep symptoms and polysomnographic patterns of obstructive sleep apnea in overweight and obese children.

**Materials & Methods:**

Overweight or obese children aging 6-18 yr old referred during 2010 to Endocrinology Clinic of Ghods Hospital in Ghazvin, central Iran were enrolled in the study. Polysomnography was done for the diagnosis of obstructive sleep apnea and the BEARS and Children’s Sleep Habits questionnaires were used to survey sleep behaviors.

**Results:**

We enrolled 30 children (14 males, 16 females). Twenty-one cases had body mass index (BMI) >95% and 9 had 85% <BMI<95%. Respiratory disturbance in polysomnography was seen in 90% of cases. Symptoms included snoring 18 (60%); frequent awakening 17 (56.6%); nocturnal sweating 15 (50%); daytime sleepiness 12 (40%); sleep talking 10 (33.3%); bedtime resistance 9 (30%); nightmares 8 (26.6%); waking up problems 6 (20%); sleep walking 6 (20%); difficult breathing 4 (13.3%); bedwetting 3 (10%) and sleep onset delay 2 (6.06%). Severe, moderate and mild apnea – hypopnea Index (AHI) were seen in 12, 9 and 6 subjects, respectively. A significant Pearson correlation was found between the BMI values and sleep latency.

**Conclusion:**

Prevalence of obstructive sleep apnea is high among overweight and obese children. Physicians should be familiar with its manifestations and consider polysomnography as an invaluable diagnostic test. There was no relation between the degree of obesity and severity of obstructive sleep apnea.

## Introduction

Sleep-disordered breathing (SDB) is a constellation of breathing disorders characterized by intermittent hypoxia, hypercapnia and sleep fragmentation. Obstructive sleep apnea hypopnea syndrome (OSAHS) is usually considered as an extreme of SDB (1). The underlying cause of SDB in children remains unclear and seems to be multifactorial. The most common cause is adenotonsillar hypertrophy but other factors such as obesity, anatomic or functional defect of upper or lower airway and genetic factors are contributed (1). The prevalence of OSAHS in obese children has been reported 13% to 59% versus 2% in general pediatric population (2, 3). Obesity predisposes children to OSAHS through the adenotonsilar hypertrophy and local fat deposition within the airway luminal walls and upper airway narrowing. In addition, it may have direct effect on respiratory muscles and ventilatory control (1). On the other hand, for children, the prevalence of obesity is continuously increasing in both developed and developing countries, besides, it is considered as a significant public health problem (4). Based on an Iranian national survey in 2007, the prevalence of overweight and obesity amongst children between 6 and 18 years was 10.1% and 4.79%, respectively (5). Children with OSAHS usually have a history of snoring, observed apneas, arousals, sweating, enuresis during sleep and restless sleep at nights as well as daytime sleepiness. Morning headaches, concentration difficulties, memory loss and educational failure are other daytime symptoms (6). Lack of early detection and treatment of OSAHS can have serious adverse effects on growth velocity, behavioral and cognitive status (7), quality of life (8), use of health care services (9) and resulting in more frequent cardiovascular and chronic illness comorbidities (10). Therefore, it is mandatory to monitor the symptoms and diagnosis of OSAHS in the suspicious children such as obese and overweight ones. Polysomnographgy (PSG) that consists of a concurrent recording of multiple physiologic parameters related to sleep and wakefulness is often considered as the gold standard test for the diagnosis of OSAHS that determine the severity of the disease. PSG can directly detect and quantify the number of respiratory events and the resultant hypoxemia as well as arousals related to the respiratory events. Although obesity has been recognized as a risk factor for adult OSAHS, there are more conflicts about this correlation in children. In this study, we attempted to detect the clinical symptoms and PSG pattern of obstructive sleep apnea in overweight and obese children.

## Materials & Methods

Overweight or obese children aging 6-18 years old referred to Endocrinology Clinic of Ghods Hospital in Ghazvin, central Iran during 2010 were enrolled in the study. Children with upper airway disease, perennial allergy or asthma, genetic diseases with high susceptibility to sleep apnea such as neuromuscular disease, Piere Robin or Down syndrome were excluded. Standing height was measured in millimeter using a wall-mounted stadiometer and weight through a Seca balance to the nearest 0.1 kg. The results expressed in the form of body mass index (BMI) reference charts and age-related prevalence for boys and girls. Children with 85% <BMI95% were considered overweight and obese, respectively. PSG was conducted for all children under the observation of a trained nurse. One of the parents accompanied their child through the night. The bedroom had a proper temperature and lighting, adequate ventilation, minimal sources of noise and a comfortable bed. A 6-channel computerized PSG was developed with leads for an oronasal flow cannula, a thoracoabdominal strain gauge and electromyogram, pulse oximeter, body-position sensor, electroencephalogram and static charge– sensitive bed. After calibration of the device, along with turning off lights, computers began recording data. The measured parameters included sleep onset latency, sleep efficiency, sleep stages (N1, N2, N3, REM), arousal index (AI), apnea hypopnea index (AHI), mean saturation O2 (SaO2), total sleep time and waking after sleep onset. The BEARS questionnaire was used in children with PSG evidences of OSAHS to investigate their sleep symptoms. This questionnaire surveys five basic sleep domains; B: bedtime problems; E: excessive daytime sleepiness; A: awakenings during the night; D: duration and regularity of sleep/wake cycles; and S: snoring (12). The Persian version of this questionnaire is prepared and its validity and reliability is confirmed (13). The second questionnaire we used for more details was the Children’s Sleep Habits Questionnaire (CSHQ). Parents were asked to recall the sleep behavior of their child over a typical week. CSHQ consists of 33 items in eight subscales about sleep duration, bedtime resistance, sleep-onset delay, daytime sleepiness, night awakenings, parasomnias, sleep-disordered breathing, and sleep anxiety. This questionnaire is also translated into Persian (14,15). Collected data were scored and analyzed by a pediatric sleep specialist using the American Academy of Sleep Medicine criteria for children (16). Apnea-hypopnea index (AHI) - which expresses the number of breathing abnormalities per hour during sleep - was used to assess the severity of sleep apnea. Apnea defined as the absence of both nasal and oral airflow, and hypopnea as a reduction of 50% or more in the airflow both lasting for more than two respiratory cycles. Respiratory events for at least 10 seconds accompanying by at least a 3% decrease in oxygen saturation and/or an associated arousal were considered significant. Obstruction was defined as the presence of apnea with continuous respiratory effort recorded by thoracoabdominal strain gauge and electromyogram. A central apnea was defined as the absence of both the airflow and breathing efforts. Mixed apneas begin with a central component and end with an obstructive one (17). The number of apneas/hypopneas event/h was used to evaluate the severity of OSAHS as divided into mild, moderate and severe if AHI number was 1-4, between 5-10 and >10, respectively (16). Data were presented as mean and standard deviation. The relationship between the SDB variables and obesity values was analyzed using Pearson correlation coefficients. A written informed consent was taken from parents. The study was approved by the Ethics Committee of Tehran University of Medical Sciences, Tehran, Iran.

## Results

**Fig 1:**
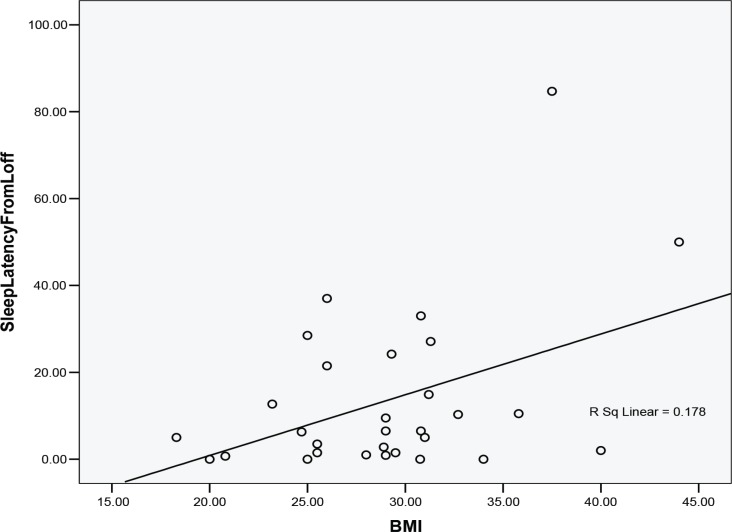
Correlation between the BMI values and sleep latency (0.421) (P=0.02)

Overall, 30 children (14 males, 16 females) were enrolled. The overall mean age was 11.18±2.87 yr (6- 17 yr). Twenty-one cases had BMI>95% and 9 had 85% <BMI<95%. The mean BMI was 26.18±10.50%. Based on questionnaires, children slept 9h, 25 min+/- 1h, in weekdays and 10 h, 27 min+/-1 h, 51 min at the weekend. Sleep symptoms of OSAHS included snoring 18 (60%); frequent awakening 17 (56.6%); nocturnal sweating 15 (50%); daytime sleepiness 12 (40%); sleep talking 10 (33.3%); bedtime resistance 9 (30%); nightmares 8 (26.6%); waking up problems 6 (20%); sleep walking 6 (20%); difficult breathing 4 (13.3%); bedwetting 3 (10%) and sleep onset delay 2 (6.06%) subjects. PSG monitoring: Total recording time was 457.56± 60.61 minutes and total sleep time 435.93±55.51 min with sleep efficiency (the amount of efficient sleep) of 75.71± 15.42 % and sleep latency of 13.57±18.65 min. Sleep architecture consisted of 10.20±6.64% stage N1, 49.80± 9.34% stage N2, 20.49±9.74% stage N3 (three stages of non REM sleep) and 11.80±4.92% rapid-eyemovement (REM) sleep. AI was 19.86±20.22/h (Shortterm awakenings at night with 3-15 seconds duration that occurred spontaneously or due to respiratory events, snoring or body movements). AI was normal in 11 and more than 11/ h in 19 subjects. Table 1 shows the sleep structure in obese children. Severe, moderate and mild AHI were seen in 12, 9 and 6 subjects respectively. The patients’ hypopnea index was 16.30±17.36/hour. The specific obstructive AHI was measured 33.13±52.20/h, central AHI 3.53±3.62/h and mixed AHI 1.96±3.85/h. The patients’ overall respiratory disturbances index (RDI) was 12.55±21.57/h. The mean baseline arterial oxygen saturation was 96.77± 1.29% and 95.3±5.7% during awaking and REM sleep stage, respectively. The lowest arterial oxygen saturation was 87.43 ± 6.7%. Table 2 shows the breathing indices in obese children. There was a significant Pearson correlation between the BMI values and sleep latency (0.421) (P=0.02) (Figure 1) but no correlation was seen between BMI values and total sleep time, sleep efficiency, AHI, RDI, arousal index and average O2sat during REM.

**Table 1:** Sleep Structure of Obese Children

	**Minimum**	**Maximum**	**Mean**	**Std. Deviation**	**Variance**
**Sleep Efficiency (%)**	41.10	97.50	75.7100	15.42596	237.960
**Sleep Latency (minutes)**	0.00	84.70	13.5700	18.65962	348.181
**N1 stage (%)**	0.90	27.70	10.2000	6.64379	44.140
**N2 stage (%)**	28.10	72.00	49.8067	9.34499	87.329
**N3 stage (%)**	4.00	42.60	20.4933	9.74807	95.025
**REM stage (%)**	0.50	20.60	11.8000	4.92635	24.269
**Arousal Index (time/hour)**	0.00	64.10	19.8667	20.22444	409.028

**NI stage: **The first stage of non- REM sleep

**N2 stage: **The second stage of non- REM sleep

**N3 stage: **The third stage of non- REM sleep**REM stage: **rapid-eye-movement stage of sleep

**Table 2.** Breathing indices of obese children

	**Minimum**	**Maximum**	**Mean**	**Std.** **Deviation**	**Variance**
**Apneas Total central (times/h)**	0.00	00.14	3.5333	3.62685	13.154
**Apneas Total mixed (times/h)**	0.00	00.18	1.9667	3.85499	14.861
**Apneas Total obstructive (times/h)**	0.00	00.270	33.1333	52.20763	2725.637
**Hypopneas Total (times/h)**	0.00	53.00	16.3000	17.36852	301.666
**Total Events RDI (times/h)**	0.20	93.90	12.5567	18.70581	349.907
**AHI Total Back (times/h)**	0.00	240.00	24.2267	44.92667	2018.405
**Average o2 While Awake (%)**	92	98.80	96.7733	1.29640	1.681
**Average o2 While in REM (%)**	65.90	99.20	95.0367	5.76078	33.187
**Approximate Minimum o2 value (%)**	73	96	87.4333	6.72455	45.220

## Discussion

Cross-sectional studies around the world revealed short sleep duration might be associated with the development of obesity in children, which is the underlying factor for sleep apnea. The sufficient sleep duration recommended for children is varied regarding their age groups (18). In the current study, sleep duration based on questionnaires was 9 h and 25 min. Iranian children have shorter night sleep duration than expected to normal ranges for their age group (11, 19). This may be due to cultural sleeping habits and poor sleep hygiene (14). Short sleep duration results in daytime sleepiness and fatigue and decreased physical activity. Furthermore, it affects the body metabolism including immune and endocrine systems function, particularly glucose and insulin metabolism that induces changes in the center of appetite followed by increased need to eat and calorie intake (moraba 20). Both of these mechanisms lead to increased BMI that predispose children to sleep apnea. Regarding the major signs and symptoms, different prevalence of sleep problems has been reported (21, 22). Similar to the report of American Thoracic Society that dyspnea, restless sleep and night sweats were the other common symptoms, respectively, snoring was the most prevalent symptom of OSAHS in our study (23). Overweight and nocturnal enuresis in children were significantly associated with OSAHS especially when they display other accompanied symptoms (24). In addition, Brooks et al. showed higher risk for enuresis in children with RDI>1 and pertained it to the effects of obstructive sleep apnea on arousal response, bladder pressure, or urinary hormone secretion (25). The prevalence of bedwetting in our study was much lower that may be partly due to the upper age group of children. In contrast, nocturnal sweating was found in 50% of our children that propound it as a diagnostic factor. Half of OSA patients had nocturnal sweating in the neck and upper body area (26). Arnardottir et al. showed threefold higher rate of nocturnal sweating in OSA patients comparing general population and stated it is related to low REM sleep percentage, lower sleep quality and more daytime sleepiness in patients. They propounded nocturnal sweating as a marker of increased risk of OSA in general population (27). Nocturnal sweating may be due to labored breathing (20), however, causes, evaluations and management of nocturnal sweating are still of matter of controverting (28). In a study, daytime sleepiness was observed in 13.5% of 37 obese children, which was much lower than our study (29). Excessive daytime sleepiness is a cardinal symptom in adolescents but hyperactivity or inattention is more obvious in preadolescent with OSAS (20). Refaey et al. (30) reported the mean sleep efficacy and sleep latency in normal weight children as 83.46 and 39.06, while our results in obese children were much lower in both items. In addition, arousal index in our study was much higher than that of their normal group (19.86/h against 1.62/h). The lowest oxygen saturation in our study was 87.43, which was much lower than reported earlier as 91.4% (30). However, sleep architecture reported there (30), as the mean REM and NREM as 8.6% and 92.6% in normal children were similar to our findings (11.80% and 80.40%). Respiratory events in children more than 1/h, is abnormal and can lead to the clinical signs and symptoms (daily growth failure, attention deficit, behavioral disorders etc.) due to changes in sleep architecture and arterial oxygenation that affect prefrontal executive function (48 moraba). The mean RDI in our study was 12.55/h, which is much higher than 1.37/h reported in normal children (30). It seems that our results are similar to the report of Dayyat et al. in 105 obese children aged over 7 yr as the mean oxygen saturation was 92.95±10.5%, arousal index 15.89±2.8/h and RDI 9.1± 1.1/h (31). In obese children, airway size is reduced and its resistance is increased in the favor of excessive fat deposition around the upper airway. In addition, lung volumes and central ventilatory drive both are reduced in obese children that lead to more decrement of the upper airway patency (20). Obesity is the most important risk factor for OSAS in 2 – 18 yr old children, with an odds ratio of 4.5 (32). In the current study, 90% of children had OSAHS in PSG. Compared to similar studies, this rate is much higher, which could be due to our methodological limitations. Although our subjects were enrolled from an obesity clinic and not from an otolaryngology or sleep clinic, however, presumably only children with suspected OSAHS were referred. The great limitation of this study is the lack of a control normal weight population for comparing with obese children. In addition, despite PSG is the standard test for the diagnosis of OSAHS, the high cost and lack of parental consent resulted in limited number of our samples.

**In conclusion**, prevalence of OSAHS is high among overweight and obese Iranian children. Physicians should be familiar with the related manifestations and consider PSG as an invaluable diagnostic test. We did not find any relation between the degree of obesity and severity of OSAHS but A significant Pearson correlation was found between BMI values and sleep latency (0.421). Monitoring the symptoms of sleep apnea in obese children is recommended and if possible, PSG should be done for confirmation. Therefore, proceeding programs to increase the knowledge of parents and physicians is suggested.
